# A new synonym from Hawaii and lectotypification of *Plagiothecium longisetum* (Plagiotheciaceae)

**DOI:** 10.3897/phytokeys.164.56612

**Published:** 2020-10-21

**Authors:** Grzegorz J. Wolski, Jarosław Proćków

**Affiliations:** 1 Department of Geobotany and Plant Ecology, Faculty of Biology and Environmental Protection, University of Lodz, ul. Banacha 12/16, 90-237 Lodz, Poland University of Lodz Lodz Poland; 2 Department of Plant Biology, Institute of Biology, Faculty of Biology and Animal Science, Wrocław University of Environmental and Life Sciences, ul. Kożuchowska 7a, 51-631 Wrocław, Poland Wrocław University of Environmental and Life Sciences Wrocław Poland

**Keywords:** Codes of Botanical Nomenclature, *Orthophyllum* section, *
Plagiothecium
*, *Plagiothecium mauiense*, *Plagiothecium nemorale*, typification

## Abstract

*Plagiothecium mauiense* was first described in 1927 by V.F. Brotherus, based on materials from Hawaii. It has, so far been, treated as a separate species. A detailed analysis of the original material housed in the New York Botanical Garden Herbarium (NY01256708) found the specimen to be characterised by a lack of metallic lustre; concave, asymmetrical, lanceolate to lanceolate-ovate leaves, shrunken in their dry condition; a straight, not denticulate, acute to apiculate apex; elongate-hexagonal cells in irregular transverse rows, 101–131 × 15–21 µm at mid-leaf; very lax areolation, with decurrencies composed of three rows of cells. These characteristics indicate that this species is identical to the original material of *P. longisetum* (e.g. H-SOL 1563 011; PC0132572). Hence, we propose that *P. mauiense* should be recognised as a new synonym of *P. longisetum*. In addition, a review of *P. longisetum* syntypes found one (H-SOL 1563 011) to have the same date of collection as the protologue, and to possess a quite abundant gametophyte turf with well-preserved sporophytes, indicating it to be fertile. Considering the above, we propose that specimen H-SOL 1563 011 be designated the lectotype of *P. longisetum*.

## Introduction

In this paper we demonstrate that all the characteristics of the original material of *P. mauiense* Broth. are identical to those of the *P. longisetum* Lindb. type. Hence, we propose *P. mauiense* as a new synonym of *P. longisetum*. In addition, among the three syntypes of *P. longisetum*, we propose the specimen (H-SOL 1563 011) deposited at the Herbarium of the University of Helsinki (Finland) as the lectotype of this name. The aim of the work is to demonstrate that *P. mauiense* and *P. longisetum* are synonyms, and to propose a lectotype for the name of the latter.

The study was based on herbarium specimens analysed during research conducted at the NY Herbarium (The New York Botanical Garden, New York, U.S.A.) from November to December 2018 and November to December 2019, as well as at the PC Herbarium (Muséum National d’Histoire Naturelle, Paris, France) in January 2019; and on specimens loaned from the H Herbarium (The University of Helsinki, Helsinki, Finland). However, due to renovation ongoing at the S Herbarium (The Swedish Museum of Natural History, Stockholm, Sweden) and the temporary closure of some herbaria, some less important specimens could not be examined; despite this, they are cited in the manuscript to present the most complete dataset possible. Specimens that were analysed directly were marked with an exclamation mark.

## *Plagiothecium mauiense* account

In *Hawaiian Mosses*, V.F. Brotherus described a new species, *Plagiothecium mauiense*, based on materials collected by D.D. Baldwin from Hawaii (Brotherus 1927). In the diagnosis, the author indicated that, among others, the plant was relatively large, soft with a thick, light green to yellow green turf (“*robustiusculum, caespitosum, caespitibus, densiusculis, mollibus, lutescenti-viridibus*”); with loosely-arranged and complanate-foliate (“*laxiscule et complanate foliosus*”), decurrent (“*folia haud decurrentia*”), concave (“*concaviuscula*”), asymmetrical (“*asymmetrica*”), long-ovate leaves (“*ovate-oblong* [sic.]”); the leaf apex was short, acute to acumiante (“*breviter acumianta, acuta vel subula brevissima terminata*”); the leaves were 2.25 mm long and 1.1 mm wide (“*ad 2.25 mm longa et ad 1.1 mm lata, integra*”), costae were short and thin (“*nervis binis, brevibus, tenuibus*”); cells at mid-leaf are 12–15 × 75–100 µm (“*cellulis medianis folii 12–15 µm longis et 75–100 µm latis*”) (Brotherus 1927). Additionally, Brotherus (1927) added that the species *P. mauiense* was similar to *P. sylvaticum* (Brid.) Schimp., however, its cell areolation was narrower.

**Figure 1.**
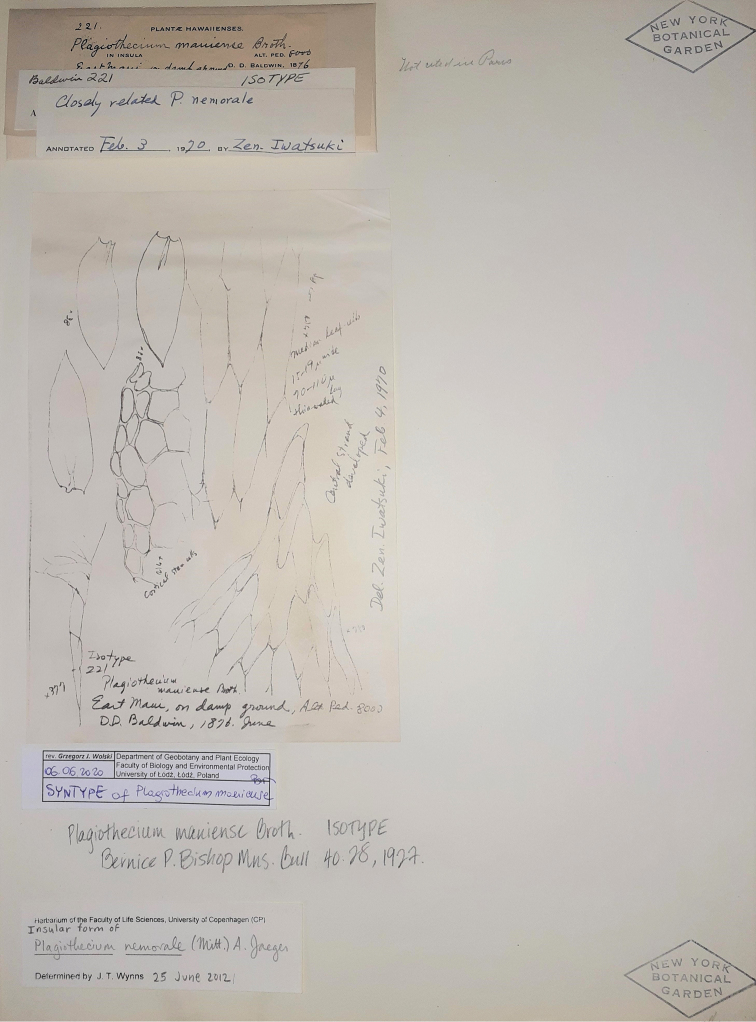
The syntype of *P. mauiense* (NY01256708) housed at the NY Herbarium.

During the revision of *Plagiothecium nemoralesensu lato*, the original materials collected by D.D. Baldwin from Hawaii were found in four herbaria: Harvard University Herbarium (FH00220142), New York Botanical Garden Herbarium (NY01256708), Miami University Herbarium (MU 000000546), and Yale University Herbarium (YU 233890). On the envelopes of two specimens, from the MU and NY Herbaria, notes indicating them to be isotypes of *P. mauiense* were also found. In 1967, a similar note was added to the specimen from the NY Herbarium (NY01256708) by H.A. Miller, who studied this material (Fig. 1). Since that time, this specimen has served as the “isotype” (e.g. in the database of the Consortium of North American Bryophyte Herbaria, https://bryophyteportal.org/portal/ – access: May 2020).

However, given the above, and according to Article 9.6 of the *Shenzhen Code* (Turland et al. 2018) stating that “A syntype is any specimen cited in the protologue when there is no holotype, or any one of two or more specimens simultaneously designated in the protologue as types” as well as Article 40.2 (Note 1) stating that “When the type is indicated by reference to an entire gathering, or a part thereof, that consists of more than one specimen, those specimens are syntypes (see Art. 9.6.)”, all the above-mentioned original specimens of *P. mauiense* should be regarded as syntypes.

Three years later, on April 3, 1970, following a study by H.A. Miller, Z. Iwatsuki analysed the same material (NY01256708) and attached a note (dated April 4, 1970) to the examined specimen, together with hand-drawn pictures of its gametophyte (Fig. 1). Based on the remaining notes, it appears that Iwatsuki characterised the material as follows: stems with a developed central strand; leaves rather asymmetrical; decurrencies composed of one row; the apex very slightly denticulate; thin-wall cells in irregular transverse rows, at mid-leaf 15–19 × 90–110 µm. Additionally, Iwatsuki left a note that *P. mauiense* is closely related to *P. nemorale* (Mitt.) A. Jaeger (Iwatsuki 1970 unpubl.). However, in an analysis of *P. nemorale* in a taxonomic revision of the genus *Plagiothecium* published the same year, Iwatsuki (1970) does not mention *P. mauiense* or its similarity to this species.

Over 40 years later (June 25, 2012), the specimen deposited at the New York Herbarium (NY01256708) was reviewed by J.T. Wynns, who added the note: “Insular form of *Plagiothecium nemorale* (Mitt.) A. Jaeger”. In addition, in his revision of the genus *Plagiothecium*, he also added next to *P. mauiense* that the material did not differ from the type of *Stereodon nemoralis* Mitt. (being a basionym of *P. nemorale*) (Wynns 2015).

Despite the above-presented assumptions indicating that *P. mauiense*, recorded from Hawaii, is closely related or even identical to *S. nemoralis*, the two are still treated as separate species (see: Hoe 1974; Staples et al. 2004).

The features given in the diagnosis by Brotherus (1927), and indicated by Iwatsuki (1970 unpubl.) based on the analysis of the original material (Fig. 1), clearly qualify the described collections as a taxon representing the genus *Plagiothecium* and belonging to the section Orthophyllum Jedl. They even classified it as belonging to *P. nemoralesensu lato.* However, as some of the features given by Brotherus and Iwatsuki contradict each other, particularly the most taxonomically significant one, i.e. the length of the cells of the central part of the leaves, it is impossible to clearly assess this material.

**Figure 2.**
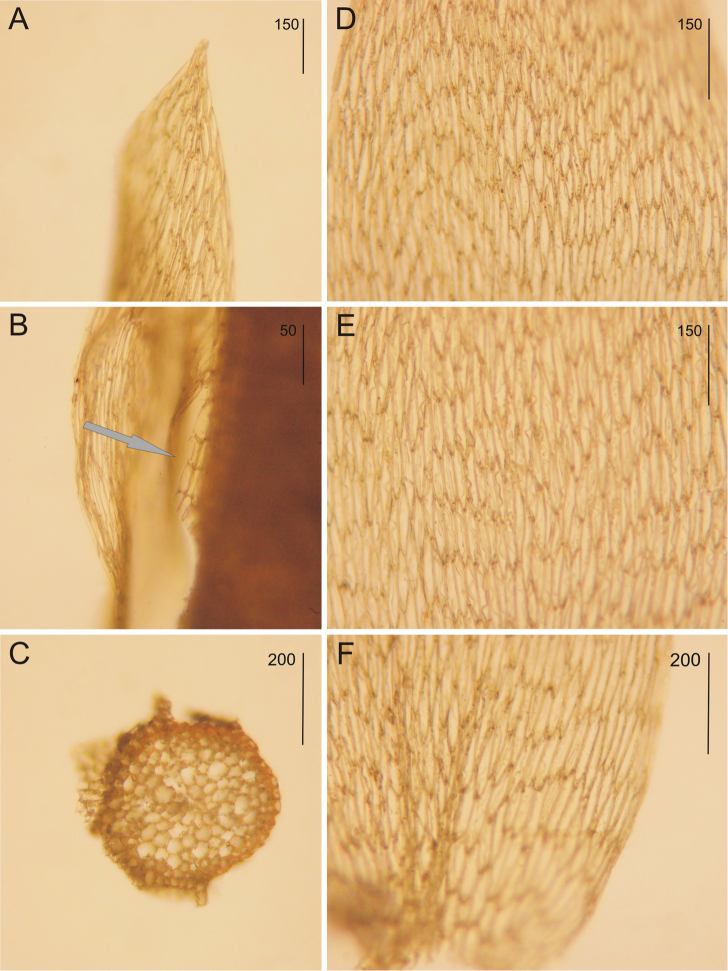
*Plagiothecium mauiense* from the NY Herbarium (*D.D. Baldwin 221*, NY01256708) **A** the plain leaf apex **B** the grey arrow indicates the three rows of decurrencies **C** the stem cross section **D–F** the shape and dimensions of cells from individual leaf zones: **D** from the upper part **E** from the middle part **F** from the lower part of the leaf. Scale bars: 50 µm (**B**); 150 µm (**A, D, E**); 200 µm (**C, F**).

Its light green to yellow green turf colour, leaf asymmetry and narrow cell areolation and irregular arrangement of cells reported by Brotherus (1927) and Iwatsuki (1970 unpubl.) are all characteristic of *P. longisetum*; in addition, Iwatsuki (1970 unpubl.) described the presence of a denticulate apex, corresponding to that of *P. nemorale*. Most importantly, the two authors differ in their opinion of the cell length at mid-leaf, one of the most taxonomically important features of this genus: Brotherus (1927) reported the length to be 75–100 µm, which clearly matches *P. nemorale*, while Iwatsuki (1970 unpubl.) reported it as 90–110 µm, corresponding to *P. longisetum*. In addition to the leaf cells, another very important feature of the whole genus, which is characteristic of individual sections, is the nature of leaf decurrencies (e.g. Nyholm 1965; Smith 2001; Wynns et al. 2017). Iwatsuki (1970 unpubl.) reported the presence of a single row of decurrent cells, which is a feature shared with representatives of section Leptophyllum Jedl. rather than section Orthophyllum, which is characterised by 2–3 rows of decurrent cells (Figs 1, 2). The remaining set of features provided by both authors are characteristic of both species: a large plant with a thick turf; loosely arranged and complanate-foliate; large (2.25 mm long and 1.1 mm wide), concave, long-ovate leaves; two costae; an acute to acuminate apex; a developed central strand; thin-wall cells (Brotherus 1927; Iwatsuki 1970 unpubl.; Wolski 2017, 2018, 2020; Wolski and Nowicka-Krawczyk 2020).

The above-presented features and a detailed analysis of the specimen deposited at the New York Herbarium (NY01256708) indicated that this material represents *P. longisetum*. The specimen is large, light green to yellowish green, without metallic lustre; stems up to 2–2.5 cm long, complanate-foliate, rounded in cross-section, 330–380 µm in diameter, a developed central strand, epidermal cells 7–16 × 14–24 µm, parenchyma thin-walled, 22–47 × 19–43 µm; leaves gently concave, asymmetrical, lanceolate to lanceolate-ovate, spreading, shrunken in dry conditions, those from the middle of the stem 2.5–2.7 mm long, and 1.1–1.5 mm in width, measured at the widest point; the apex straight, not denticulate, acute to apiculate; two costae, extending almost to ½ leaf length, reaching 0.50–0.70 mm; elongate-hexagonal cells in irregular transverse rows, areolation very lax; cells reach 85–134 × 15–20 µm at the apex, 101–131 × 15–21 µm at mid-leaf, and 113–170 × 18–25 µm at the lower part of the leaf; decurrencies of three rows of rectangular cells, 32–44 × 15–31 µm (Fig. 2).

As only one syntype was examined (NY01256708), and due to the current inability to examine specimens from the other three herbaria (from FH, MU, YU), lectotypification of *P. mauiense* will be carried out later, once all original materials collected by D.D. Baldwin from Hawaii have been examined.

## *Plagiothecium longisetum* account

**Figure 3.**
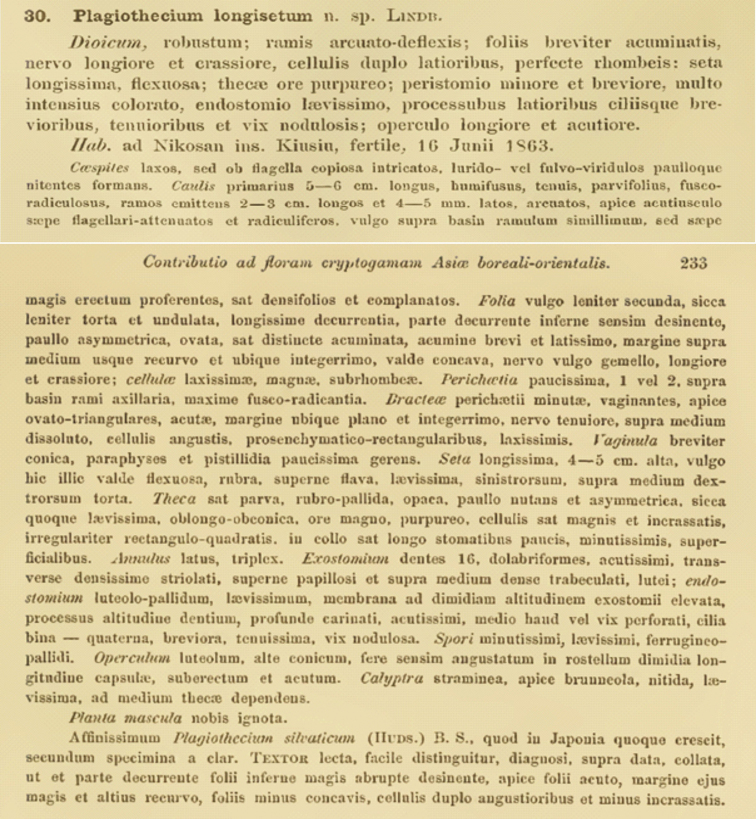
The diagnosis of *Plagiothecium longisetum* (Lindberg 1872).

Lindberg described *Plagiothecium longisetum* in *Contributio ad Floram Cryptogamam Asiae Boreali*-*Orientalis* based on materials collected by C. Maximovicz from Japan (Lindberg 1872) (Fig. 3). After this fact, in the 19^th^ and 20^th^ centuries, the species was noted in the most important bryological studies of that time (Jaeger 1875–1876; Paris 1894–1898), however, it was not given from Europe, and its range was limited only to East Asia (China and Japan) (Schimper 1876; Lindberg 1879; Gravet 1883; Mitten 1891; Kindberg 1897; Brotherus 1929; Grout 1932; Podpéra 1954; Sakurai 1954).

At the beginning of the 20^th^ century, Cardot (1912) indicated a relationship between *P. longisetum* and *P. sylvaticum*, writing that the former, similarly to *P. nemorale*, was just a form of *P. sylvaticum*. Wijk et al. (1967) indicated that *P. longisetum* was a synonym of *P. sylvaticum*, whereas Iwatsuki (1970) wrote that *P. longisetum* was a synonym of *P. nemorale*, adding that the former was only a habitat modification of the latter. After Iwatsuki (1970), for the next 50 years, this view spread throughout Europe and Asia (Lewinsky 1974; Iwatsuki 2004; Wynns 2015; Suzuki 2016). However, at the beginning of the 21^st^ Century, as a result of a taxonomic revision of *P. nemoralesensu lato*, Wolski and Nowicka-Krawczyk (2020) proposed the resurrection of *P. longisetum*, and for it to be treated as separate from *P. nemorale*, which also was distributed in Eurasia. Subsequent studies have revealed a number of differences between the two species in the micromorphology of their sporophyte; they also documented their presence in North America, thus extending their global range (Wolski 2020; Wolski et al. 2020).

**Figure 4.**
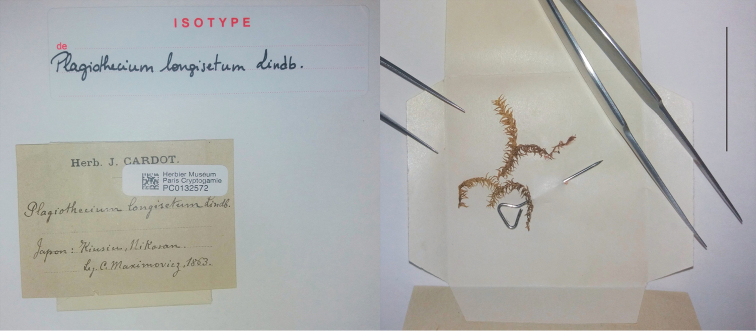
Sheet of *Plagiothecium longisetum* marked as the “isotype” and three stems of gametophyte deposited in the herbarium of the Muséum National d’Histoire Naturelle (PC0132572). Scale bar: 3 cm.

During this revision, the specimens on which Lindberg (1872) described *P. longisetum* were found in three herbaria: the University of Helsinki Herbarium (H-SOL1563011), the Herbarium of Swedish Museum of Natural History (S-B160017) and Muséum National d’Histoire Naturelle (PC0132572). The latter specimen is marked as the “isotype” (Fig. 4). Based on this information, and according to Article 9.6 and 40.2 Note 1 of the *Shenzhen Code* (Turland et al. 2018) cited above, all specimens listed above should be considered syntypes.

In addition to the description of gametophyte morphology given in the diagnosis of *P. longisetum*, Lindberg (1872) indicated that this material has sporophytes (i.e. is “*fertile*”) and was collected on 16 June, 1863 (“*16 Junii 1863*”) near Nikosan on Kyushu island (“*ad Nikosan ins. Kiusiu*”) in Japan (Fig. 3). Additionally, Lindberg (1872) indicates, among others, that *P. longisetum* is characterised by a very long seta (“*seta longissima*”) and a long operculum (“*operculo longiore et acutiore*”). The presence of such a long seta, i.e. up to 5 cm in length, and a long operculum, distinguish the sample from other species of *Plagiothecium*; these characteristics, combined with the gametophyte features, are unique to *P. longisetum* (Wolski and Nowicka-Krawczyk 2020).

**Figure 5.**
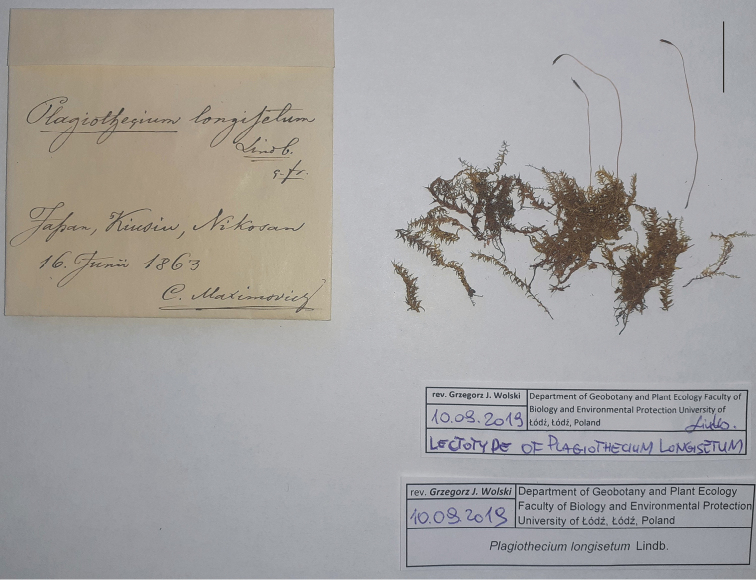
Envelope and turf with sporophytes of *Plagiothecium longisetum* from the University of Helsinki Herbarium (H-SOL 1563 011). Scale bar: 2 cm.

The specimen deposited at the herbarium in Helsinki (H-SOL 1563 011) was awarded the same date of collection as in the prologue, and is characterised by a fairly large, well-preserved gametophyte turf with three sporophytes (Fig. 5). The material deposited in Stockholm (S-B160017) also has a full collection date; however, due to the ongoing renovation of this herbarium, the loan and subsequent analysis of this material is impossible. Unfortunately, the specimen housed in the herbarium in Paris (PC0132572) has an incomplete collection date (“*1863*”), and only three gametophyte stems, without sporophytes (Fig. 4).

The material deposited in Helsinki (H-SOL 1563 011) is distinguished from other analysed syntypes by the presence of a fairly large gametophyte turf and more importantly, well-preserved sporophytes (Fig. 5), which (as Lindberg described in the diagnosis) confirm that the specimen is prolific (“*fertile*”). Based on these facts, and according to Article 9.3 of the *Shenzhen Code* (Turland et al. 2018) cited above, we propose that specimen H-SOL1563011 should be designated as the lectotype of *P. longisetum* (Fig. 5). In addition, due to the fact that the specimen from Muséum National d’Histoire Naturelle (PC0132572) was examined at the beginning of 2019, and we did not have access to the specimen deposited at the Herbarium of the Swedish Museum of Natural History (S-B160017), a request was sent to both institutions to change the status of these specimens to the isolectotype.

## Taxonomic treatment

*Plagiothecium longisetum* Lindb., Acta Soc. Sci. Fenn. 10: 232 (1875). – Lectotype (designated here): [Japan], ad Nikosan ins. Kiusiu, [fertile], 16 Junii 1863, *S.O. Lindberg s.n.* (lecto-: H-SOL 1563 011!, isolecto-: PC0132572!, S-B160017) = *P. mauiense* Broth., Bernice P. Bishop Museum Bulletin 40: 28 (1927), syn. nov. Type: [United States], Hawaii, E Maui, Haleakala, 8000 ft., in damp ravines, fertile, June 1876, *D.D. Baldwin 221* (syn-: NY01256708!, FH00220142, MU000000546, YU233890).

## References

[B1] Brotherus VF (1927) Hawaiian Mosses. Bernice P. Bishop Museum. Bulletin 40: 1–28.

[B2] Brotherus VF (1929) Symbolae Sinicae. Teil IV: Musci. Verlag von Julius Springer, Wien.

[B3] Cardot J (1912) Mousses nouvelles du Japon et de Corée. Bulletin de la Société Botanique de Genève, sér. 2. 4: 378–387.

[B4] Gravet F (1883) Enumeratio Muscorum Europaeorum – Bibliographie – Nouvelles. Revue Bryologique Paraissant Tous Les Deux Mois 2: 1–34.

[B5] Grout AJ (1932) Moss Flora of North America North of Mexico (Vol. 3). Published by the author, Newfane.

[B6] Hoe WJ (1974) Annotated Checklist of Hawaiian Mosses. Lyonia 1: 1–45.

[B7] Iwatsuki Z (1970) A revision of *Plagiothecium* and its related genera from Japan and her adjacent areas. The Journal of the Hattori Botanical Laboratory 33: 331–380.

[B8] Iwatsuki Z (2004) New catalog of the mosses od Japan. The Journal of the Hattori Botanical Laboratory 96: 1–182.

[B9] Jaeger A (1875–1876) [1877] Genera et Species Muscorum Systematicae Disposita; Seu, Adumbratio Florae Muscorum Totius Orbis Terrarum (Continuatio). Sancti Galli (St. Gallen), W. Haussknecht, 444–454.

[B10] Kindberg NC (1897) European and N. American Bryineae (Mosses). Linköping, Sweden. 10.5962/bhl.title.56766

[B11] Lewinsky J (1974) The family Plagiotheciaceae in Denmark. Lindbergia 2: 185–217.

[B12] Lindberg SO (1872) Contributio ad floram cryptogamiam Asiae Boreali-Orientalis. Helsingforsiae.

[B13] Lindberg SO (1879) Musci Scandinavici in systemate novo naturali dispositi. Upsaliae.

[B14] Mitten W (1891) On the species of Musci and Hepaticae recorded from Japan. Transactions of the Linnean Society of London 3(3): 178–179. 10.1111/j.1095-8339.1891.tb00626.x

[B15] Nyholm E (1965) Family Plagiotheciaceae Illustrated Moss Flora of Fennoscandia. II. Musci. Fascicle 5, The Botanical Society of Lund.

[B16] Paris EG (1894–1898) Index bryologicus sive, Enumeratio muscorum hucusque cognitorum adjunctis synonymia distributioneque geographica locupletissimus. Parisiis. 10.5962/bhl.title.643

[B17] Podpéra J (1954) Conspectus Muscorum Europaeorum. Nakladatelství Československé Akademie Věd, Prague.

[B18] Sakurai K (1954) Muscologia japonica. Iwanami Shoten, Tokyo.

[B19] Schimper WPh (1876) Synopsis muscorum Europaeorum: praemissa introductione de elementis bryologcis tractante (Vol. 1), Stuttgartiae.

[B20] Smith AJE (2001) The moss flora of Britain and Ireland. Cambridge University Press.

[B21] Staples GW Imada CT Hoe WJ Smith CW (2004) A revised checklist of Hawaiian mosses. Tropical Bryology 25: 35–69. 10.11646/bde.25.1.7

[B22] Suzuki TA (2016) Revised new catalog of the mosses of Japan. Hattoria 7: 9–223.

[B23] Turland NJ Wiersema JH Barrie FR Greuter W Hawksworth DL Herendeen PS Knapp S Kusber W-H Li D-Z Marhold K May TW McNeill J Monro AM Prado J Price MJ Smith GF (2018) International Code of Nomenclature for algae, fungi, and plants (Shenzhen Code) adopted by the Nineteenth International Botanical Congress Shenzhen, China, July 2017. Regnum Vegetabile 159. Koeltz Botanical Books, Glashütten. 10.12705/Code.2018

[B24] van der Wijk R Margadant WD Florschütz PA (1967) Index Muscorum, vol. 4. International Bureau for Plant Taxonomy and Nomenclature of the International Association for Plant Taxonomy. Utrecht.

[B25] Wolski GJ (2017) Morphological and anatomical variability of *Plagiothecium nemorale* in Central Poland. Herzogia 30(1): 36–50. 10.13158/heia.30.1.2017.36

[B26] Wolski GJ (2018) Are *Plagiothecium cavifolium*, *P. nemorale* and *P. succulentum* indeed variable species? Pakistan Journal of Botany 50(4): 1579–1589.

[B27] Wolski GJ (2020) Reassessing the taxonomic diversity of Plagiothecium section Orthophyllum in the North American bryoflora. Brittonia 72(3): 1–14. 10.1007/s12228-020-09631-y

[B28] Wolski GJ Bihun M Białecka B Rewicz A (2020) SEM differences in sporophyte micromorphology of *Plagiothecium nemorale* and *P. longisetum* (Plagiotheciaceae, Bryophyta). Folia Cryptogamica Estonica. [in press]

[B29] Wolski GJ Nowicka-Krawczyk P (2020) Resurrection of the *Plagiothecium longisetum* Lindb. and proposal of the new species – *P. angusticellum* PLOS One 15(3): e0230237. 10.1371/journal.pone.0230237 PMC7065767 32160254

[B30] Wynns JT (2015) Molecular phylogeny and systematic revision of the pleurocarpous moss genus *Plagiothecium*. PhD Thesis, University of Copenhagen, Denmark.

[B31] Wynns JT Munk KR Lange CBA (2017) Molecular phylogeny of *Plagiothecium* and similar hypnalean mosses, with a revised sectional classification of *Plagiothecium*. Cladistics 34(5): 469–501. 10.1111/cla.12210 34649367

